# Central and Bridge Symptoms of Internet Gaming Disorder, Social Jet Lag, and Insomnia Among College Students: Cross-Sectional Network Analysis

**DOI:** 10.2196/87919

**Published:** 2026-08-12

**Authors:** Meng Qi, Jingyu Lin, Hongjie Chen, Jiaqi Sun, Shuangjiang Zhou, Jingxu Chen

**Affiliations:** 1 Beijing Huilongguan Hospital, Capital Medical University Beijing, Beijing China; 2 Department of Psychology Chengde Medical University Chengde, Hebei China

**Keywords:** college students, insomnia, social jet lag, internet gaming disorder, network analysis

## Abstract

**Background:**

Internet gaming disorder (IGD) has become increasingly prevalent among college students, while the prevalence of insomnia and social jet lag has also gradually increased in this population. Comorbidity among these conditions is common in young adults. Nevertheless, the internal correlational architecture underlying individual symptom indicators remains unclear.

**Objective:**

This study aimed to investigate the central and bridge symptoms of IGD, social jet lag, and insomnia among college students; to explore the network structure and cross-symptom connectivity among them; and to provide evidence for targeted interventions for comorbid insomnia and IGD.

**Methods:**

This cross-sectional study targeted a national sample of Chinese college students. A total of 1346 participants were included in this study, with a mean age of 19.35 (SD 1.35) years, including 668 (49.6%) male participants and 678 (50.4%) female participants. All participants completed a general information questionnaire, the Internet Gaming Disorder Scale-Short Form (IGDS9-SF), the Munich Chronotype Questionnaire (MCTQ), and the Pittsburgh Sleep Quality Index (PSQI). Network analysis was used to investigate the network structure, identify central and bridge symptoms, quantify the correlation intensity between indicators, and assess network stability. Expected influence (EI) and bridge expected influence (BEI) were used to measure centrality.

**Results:**

Three distinct variable clusters were extracted from the overall network: the insomnia factor cluster, the IGD symptom cluster, and social jet lag. The insomnia symptom “subjective sleep quality” (P1; strength=0.903) and the IGD symptom “loss” (I9; strength=2.463) were the most central nodes in the network, constituting the key indicators of this study. The bridge strength of all nodes ranged from 0.029 to 1.295. “Withdrawal” (I2) exhibited markedly higher bridge strength than all other nodes (1.295, 95% CI 1.162-1.428), followed by “social jet lag” (S1; 0.522, 95% CI 0.384-0.690) and “preoccupation” (I1; 0.519, 95% CI 0.364-0.718). As the key bridging symptom between the insomnia and IGD clusters, I2 had the strongest edge connection with “daytime dysfunction” (P7), with an edge weight of 0.329 (95% CI 0.246-0.450). Connections involving the social jet lag cluster (S1) were concentrated on “continue despite problems” (I6; 0.182, 95% CI 0.012-0.364), while the connections between the insomnia symptom cluster and S1 were all low.

**Conclusions:**

Unlike previous studies that focused on bivariate associations or single disorders, our findings reveal that I2 serves as the key bridging symptom connecting insomnia and IGD, while P1 and I9 are central nodes within their respective structural communities. These findings provide a novel perspective on the comorbidity of insomnia and IGD. In real-world practice, interventions targeting withdrawal symptoms should be prioritized, as they may alleviate comorbid symptoms simultaneously. The role of S1 was relatively limited, suggesting that managing circadian rhythm disruption may indirectly reduce the risk of IGD. These findings provide actionable symptom targets for prevention and treatment programs in university settings.

## Introduction

### Problem

Sleep deprivation is prevalent among college students. The overall prevalence of sleep disorders among Chinese adolescents and young adults is 20.7% [1]. Moreover, the misalignment between circadian rhythms and socially mandated schedules has become common among college students and is referred to as social jet lag (SJL) [2]. This phenomenon persists throughout an individual’s academic and professional career [3]. Approximately 69% of individuals experience at least 1 hour of SJL during the work week, while nearly 33% experience 2 hours or more [4,5]. These conditions not only harm the physical health of individuals with chronic insomnia but also have a significant negative impact on their daily functioning [6]. Parallel to insomnia and SJL among college students is internet gaming disorder (IGD). IGD is primarily characterized by core manifestations, such as difficulty controlling the amount of time spent gaming, neglecting daily responsibilities and interpersonal interactions due to gaming, sacrificing academic or work opportunities to continue gaming, and experiencing damaged relationships or conflicts because of excessive gaming [7]. According to the Statistical Report on China’s Internet Development, students constitute the largest group of internet users in China, and the proportion of college students engrossed in online games is rising annually. The prevalence of IGD ranges from 0.7% to 27.5% [8]. Internet gaming addiction is increasingly eroding the physical and mental health of college students, severely impacting individual growth and development.

### Review of Relevant Scholarship

As the concept of SJL was introduced, numerous studies have demonstrated its association with various health risks, including reduced sleep quality, daytime fatigue, and depressive mood [9]. SJL causes individuals to experience a form of chronic insomnia characterized by circadian misalignment: they are forced to attempt sleep during times when their circadian rhythm favors wakefulness yet must remain awake during times when their circadian rhythm favors sleep (eg, early morning on workdays). This leads to difficulty falling asleep and daytime fatigue [10]. In response to these outcomes, some individuals adopt strategies such as smoking, alcohol consumption, and caffeine intake, which in turn reduce sleep drive and perpetuate a vicious cycle [11,12]. Furthermore, to compensate for sleep deprivation, individuals with SJL often excessively extend their time in bed on rest days. This behavioral pattern may lead to engagement in nonsleep activities in bed (eg, using electronic devices), which exacerbates insomnia at the behavioral level and may also contribute to the development of problems such as gaming addiction [13].

IGD is a significant behavioral risk factor for both the development and maintenance of insomnia, and a bidirectional relationship exists between them. Nowadays, large-scale multiplayer online battle arena (MOBA) games demand intense concentration and evoke strong emotional responses (eg, excitement, frustration, or anger). This sustained state of sympathetic nervous system arousal often persists even after the game ends [14]. Among college students, excessive engagement in online games leads to delayed bedtimes, resulting in a phase delay in sleep and disruptions to the sleep-wake rhythm [15]. Moreover, the short-wavelength blue light emitted by electronic screens suppresses endogenous melatonin secretion and disrupts the sleep-wake rhythm, leading to sleep problems [16]. Concurrently, insomnia symptoms are significant risk factors for IGD [17]. Insomnia predicts extended gaming durations, which individuals often adopt as a coping strategy for psychological distress, thereby significantly increasing the risk of developing IGD symptoms [18].

### Hypothesis, Aims, and Objectives

Although the aforementioned studies suggest pairwise associations between insomnia, SJL, and IGD, these 3 conditions often coexist in clinical practice. This may occur through circadian rhythm disruption (IGD can lead to irregular daily routines, thereby exacerbating SJL and insomnia; in turn, SJL and insomnia further reinforce problematic gaming behaviors). These 3 conditions are not independent of one another, and examining only their pairwise relationships is insufficient to fully reveal the complex interactions among them. Therefore, it is necessary to integrate insomnia, SJL, and IGD into a single network model to systematically investigate their intrinsic connections and pathways of influence at the symptom level.

Traditional research methods (eg, correlation analysis and structural equation modeling) are largely limited to examining latent variable structures of linear relationships and are inadequate for revealing the direct and indirect interaction patterns among the 3 conditions. First, it conceptualizes mental disorders as dynamic systems composed of mutually reinforcing symptoms, rather than as outcomes caused by an underlying latent disease entity [19]. Second, it focuses on direct associations between symptoms, enabling researchers to precisely identify central symptoms within the network and bridge symptoms connecting different symptom clusters, thereby revealing the maintenance mechanisms of multisymptom systems and providing more precise targets for clinical intervention [20].

In summary, this study aims to systematically explore the symptomatic associations among insomnia, IGD, and SJL based on network analysis. The specific research objectives are as follows: (1) to construct a symptom network of insomnia, IGD, and SJL to elucidate the overall pattern of associations among the 3 conditions; (2) to identify central symptoms within the overall network; and (3) to identify bridge symptoms that link the different symptom clusters, thereby providing intervention targets for clinical treatment.

## Methods

### Population, Sample, and Research Design

A convenience sampling method was adopted. From October to November 2024, a questionnaire survey was conducted among college students from partner universities using an online questionnaire platform (Wenjuanxing; WJX). This study adopted a quantitative cross-sectional design and used structured questionnaires to collect participant data. The sample size was estimated in accordance with international guidelines for symptom network analysis. A total of 17 nodes were included; on the basis of the criterion of 10 participants per indicator, a minimum of 170 participants were required for robust model construction. Considering an anticipated 10% exclusion rate for invalid questionnaires, the preliminary calculated sample size was 189 participants, and the planned recruitment size was rounded up to 1200 participants. A total of 1375 questionnaires were collected. After excluding 29 (2.11%) invalid responses, the valid response rate was 97.89% (n=1346). Among these, 21 (1.53%) questionnaires were excluded due to incomplete responses and 8 (0.58%) were excluded due to excessively short completion times. The mean age of the participants was 19.35 (SD 1.35) years, comprising 668 (49.6%) male participants and 678 (50.4%) female participants.

### Measures and Covariates

The primary outcome measures consisted of the scale scores for IGD, insomnia, and SJL. Age and sex were set as secondary indicators and covariates. Additional information, including daily routine and cognitive function, was collected concurrently during the survey; these data were not presented in the present results due to the absence of statistically significant associations with the study outcomes.

### Data Diagnostics

After questionnaire collection, invalid samples with perfunctory responses, patterned repetitive answers, or extensive missing values in the central variables were excluded. After elimination, the overall missing rate in the dataset was <5%, with only sporadic missing values for individual scale items and no cases with substantial missing data across all scales. The Little missing completely at random (MCAR) test was performed to examine the missing mechanism, yielding *χ^2^*_15_=7.1 (*P*=.96), indicating that the missing data met the assumption of MCAR. Multiple imputation was therefore adopted to handle missing values.

Observations with absolute *Z* scores exceeding 3 were defined as potential outliers. Further verification confirmed that all outlying values reflected authentic responses, and therefore all data were retained for subsequent analyses. Normality tests revealed that none of the symptom items conformed to strict normal distribution, which is consistent with the typical distribution characteristics of psychological scale data. No logarithmic transformation or standardization was applied to the raw data, and the original raw scores of all items were directly used to construct the symptom network model.

### Ethical Considerations

The study protocol was reviewed and approved by the institutional review board (IRB) of Beijing Huilongguan Hospital, Capital Medical University (2023-26-ke), which granted an exemption from full review because the study involved only noninvasive questionnaire procedures and presented no more than minimal risk to participants. All participants were university students recruited offline; prior to participation, each individual provided informed consent after being fully informed of the purpose, procedures, and voluntary nature of the study. The original informed consent explicitly permitted secondary analysis of the collected data for research purposes; therefore, no additional consent was required for the present analyses. To protect privacy and confidentiality, all data were collected anonymously using an online questionnaire platform, and no personal identifiers (eg, names, student ID numbers, or IP addresses) were recorded. No images or data that could identify individual participants are included in the manuscript or supplementary materials. Participants did not receive any form of financial compensation.

### Measures

#### Demographic Data

A self-designed general information questionnaire was used to collect participants’ demographic characteristics and basic clinical information. The questionnaire primarily included items on sex, age, academic performance, nicotine and alcohol consumption, perceived stress levels, and the presence or absence of mental or psychological disorders.

#### Internet Gaming Disorder Scale-Short Form

The diagnoses identified by this scale align with the *International Statistical Classification of Diseases and Related Health Problems, 11th edition* (ICD-11) criteria for predominantly online gaming disorder [21]. The scale consists of 9 items corresponding to the 9 core symptoms of IGD. Higher total scores indicate greater severity of IGD. The internal consistency coefficient (Cronbach α) for this scale was 0.80 in the present study [22]. According to Lemmens et al [23], adolescents who endorsed “yes” on ≥5 items of this scale were classified as having IGD [23].

#### Munich Chronotype Questionnaire

This questionnaire was used to assess SJL and average weekly sleep duration. The Munich Chronotype Questionnaire (MCTQ) collects comprehensive information on participants’ weekly schedules, including sleep patterns on workdays and free days. SJL was calculated as the absolute difference between the average midsleep time on free days (MSF) and the average midsleep time on workdays (MSW), expressed as |MSF–MSW| [3].

#### Pittsburgh Sleep Quality Index

This index assesses sleep quality over the preceding month. Items are scored on a 4-point scale ranging from 0 to 3, with higher total scores indicating poorer sleep quality. A global Pittsburgh Sleep Quality Index (PSQI) score of ≤7 was defined as good sleep quality, while a score of >7 was defined as poor sleep quality [24]. The Cronbach α coefficient for this scale among Chinese college students was 0.66 [25].

### Data Analysis

In the descriptive statistical analysis, normally distributed data were presented as mean (SD), while nonnormally distributed data were expressed as median (IQR). Statistical analyses were performed using SPSS (version 25.0; IBM Corp). Statistical significance was set at *P*<.05.

Network analysis was used to explore the central symptoms of IGD, SJL, and insomnia, as well as the associations among specific symptoms of these 3 conditions. The association network model was constructed and analyzed using JASP (Jeffreys’ Amazing Statistics Program; version 0.19.3.0) software. The network model was estimated using the Gaussian graphical model (GGM), with associations between nodes represented by partial correlation coefficients. Model selection and network sparsification were performed through least absolute shrinkage and selection operator (LASSO) regularization combined with EBICglasso. Each symptom was represented as a node, and the connections between nodes represented partial correlation relationships, with thicker lines indicating stronger associations.

Three centrality indices were visualized: strength centrality, closeness centrality, and betweenness centrality [26]. Strength centrality is defined as the sum of the absolute weights of all edges connected to a node; higher values indicate greater importance of the node within the network. Bridge centrality indices reflect the role of nodes in connecting symptom clusters of different disorders, including bridge strength centrality, bridge closeness centrality, and bridge betweenness centrality. This study used the definitions of betweenness and closeness proposed by Opsahl et al [27] for weighted networks. To identify connecting nodes among the 3 symptom clusters—insomnia, SJL, and IGD—we performed a bridge symptom analysis using the *bridge* function in R (version 4.2.3; R Foundation for Statistical Computing), based on the bridge centrality indices described previously. For each node, we computed its bridge strength, defined as the sum of the absolute edge weights between that node and all nodes belonging to other symptom clusters. Greater bridge strength values reflect a more crucial role of the node in cross‑symptom transmission. We also assessed the network stability and accuracy using the *Bootnet* package in R [28]. A total of 5000 bootstrap samples were generated to estimate the 95% CIs for the edge weights and to calculate the correlation stability (CS) coefficient. A CS coefficient of 0.25 is considered acceptable, while values >0.5 are preferable [28]. In general, symptoms with higher centrality are more central within the network and exhibit more frequent and stronger connections with other symptoms than do symptoms with lower centrality. These measures are crucial for identifying the symptoms that may potentially drive the psychopathology network.

## Results

### Characteristics of Participants

A total of 1346 college students were included in the final analysis. The participants had a mean age of 19.35 (SD 1.35) years, and 678 (50.4%) were female. Screening results indicated that 70 (5.2%) participants reported mental health issues, 546 (40.56%) had insomnia problems, and 246 (18.28%) were identified with IGD, as detailed in Table 1.

**Table 1:** Characteristics of the study participants (N=1346).

Variables		Participants
Age (years), mean (SD)		19.35 (1.35)
**Sex,** **n (%)**		
	Male	668 (49.6)
	Female	678 (50.4)
**Education, n (%)**		
	Freshman	530 (39.38)
	Sophomore	315 (23.4)
	Junior	329 (24.44)
	Senior	167 (12.41)
	Fifth year of college	5 (0.37)
**Smoking, n (%)**		
	Yes	40 (2.97)
	No	1306 (97.03)
**Drinking, n (%)**		
	Yes	201 (14.93)
	No	1145 (85.07)
**Academic achievement (%), n (%)**		
	0-20	328 (24.37)
	20-40	293 (21.77)
	40-60	417 (30.98)
	60-80	171 (12.7)
	80-100	137 (10.18)
**Mental disorders, n (%)**		
	Yes	70 (5.2)
	No	1276 (94.8)
**Insomnia, n (%)**		
	Yes	546 (40.56)
	No	800 (59.44)
**Internet gaming disorder, n (%)**		
	Yes	246 (18.28)
	No	1100 (81.72)

### Network Estimation and Visualization

**Figure 1:**
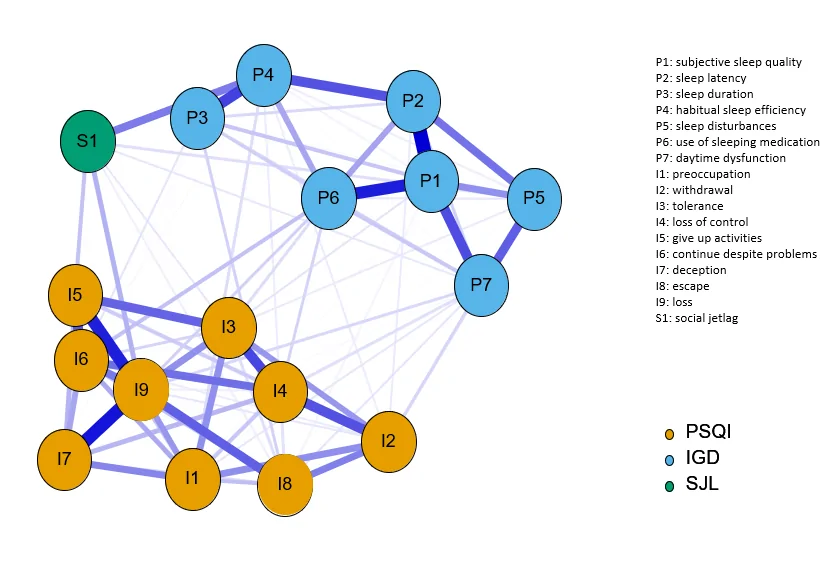
Network of internet gaming disorder (IGD), insomnia symptoms, and social jetlag (SJL) among college students. Nodes with stronger correlations are positioned closer to each other. The thickness of the edges indicates the strength of the association. Blue lines represent positive connections. The circular pie charts surrounding each node depict the predictability of the respective symptom. PSQI: Pittsburgh Sleep Quality Index.

The network of insomnia, IGD, and SJL is depicted in Figure 1. This study constructed a correlation network comprising 17 nodes (insomnia symptoms: P1-P7; IGD symptoms: I1-I9; SJL: S1; Table 2). The network had 136 possible edges, of which 78 (57.4%) were nonzero edges. The network sparsity was 0.426, indicating that the connections between nodes demonstrated moderate selectivity.

**Table 2:** Pittsburgh Sleep Quality Index, internet gaming disorder, and social jet lag network analysis centrality indices.

Variables	Item content	Betweenness (betweenness centrality)	Closeness (closeness centrality)	Strength (node strength)
P1	Subjective sleep quality	0.467	−0.058	0.903
P2	Sleep latency	0	−0.457	0.239
P3	Sleep duration	−1.052	−1.333	−1.489
P4	Habitual sleep efficiency	1.636	0.093	−0.163
P5	Sleep disturbances	−1.052	−1.95	−0.961
P6	Use of sleeping medication	0.818	0.497	−0.074
P7	Daytime dysfunction	0	−0.915	−0.257
I1	Preoccupation	−0.935	−0.647	0.249
I2	Withdrawal	0.117	−0.18	−0.237
I3	Tolerance	−0.467	−0.372	0.686
I4	Loss of control	−0.818	−0.26	0.445
I5	Give up activities	−0.701	0.965	0.402
I6	Continue despite problems	0.351	1.451	0.692
I7	Deception	−1.052	0.275	−0.415
I8	Escape	−0.467	−0.055	−0.485
I9	Loss	2.454	2.181	2.463
S1	Social jet lag	0.701	0.767	−1.998

### Centrality Indices

**Figure 2:**
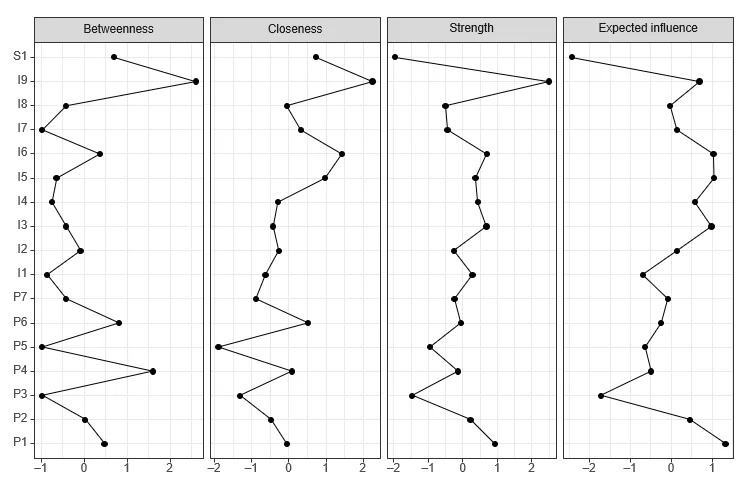
Centrality indices for nodes in the comorbidity network of internet gaming disorder, insomnia, and social jetlag.

Among the network centrality metrics, strength centrality was used as the primary indicator because it reflects the centrality of a node within the network and its connection strength. The insomnia symptom “subjective sleep quality” (P1; strength=0.903) and the IGD symptom “loss” (I9; strength=2.463) emerged as the most central nodes in the network (Figure 2), as supported by the centrality indices. The IGD symptoms “giving up other activities” (I5; strength=0.402) and “continue despite problems” (I6; strength=0.692), along with the insomnia symptom “sleep latency” (P2; strength=0.239), demonstrated relatively high network centrality. In contrast, the insomnia symptom “daytime dysfunction” (P7; strength=−0.257), the IGD symptom “escape” (I8; strength=−0.485), and SJL (S1; strength=−1.998) exhibited negative strength centrality. Closeness centrality served as a supplementary indicator, showing that I9 (2.180), I6 (1.451), and I5 (0.965) had relatively high values, while nodes such as P1 (−0.058) and P2 (−0.457) exhibited relatively low closeness centrality. Betweenness centrality was reported as an additional indicator, with results showing that the withdrawal symptom (I9; strength=2.454) and the delayed sleep onset symptom (P4; strength=1.636) had the highest values, whereas most nodes had betweenness centrality values of 0 or negative, suggesting that these items may play a role in information transmission within the network.

To facilitate comparisons, all centrality indices were standardized as *Z* scores with a mean of 0 (SD 1). Under this standardized scale, higher values indicate that a node is more central within the network, has stronger connections with other nodes, or lies on shorter paths, whereas lower values (including negative ones) reflect relatively lower node importance [29]. Therefore, the interpretation of centrality metrics in this study is primarily based on their relative magnitude, with negative values used only to characterize the degree of node peripherality without providing directional theoretical explanations.

### Bridge Symptom Analysis

The results of the bridge strength analysis are presented in Table 3. The bridge strength values across all nodes ranged from 0.029 to 1.295. The IGD item I2 exhibited the highest bridge strength (1.295, 95% CI 1.162-1.428), exceeding those of the other nodes, followed by S1 (0.522, 95% CI 0.384-0.690) and I1 (0.519, 95% CI 0.364-0.718). Within the insomnia community, P6 had the highest bridge strength (0.421, 95% CI 0.312-0.583), followed by P7 (0.402, 95% CI 0.282-0.572) and P5 (0.397, 95% CI 0.271-0.539). These findings suggest that I2 is a key bridging symptom linking IGD and insomnia, while SJL and I1 also play important roles in cross-symptom connectivity.

**Table 3:** Bridge strength of each node across insomnia, internet gaming disorder (IGD), and social jet lag communities. Full item descriptions for each node are displayed in Table 2.

Nodes	Community	Bridge strength (95% CI)
I2	IGD	1.295 (1.162-1.428)
S1	Social jet lag	0.522 (0.384-0.690)
I1	IGD	0.519 (0.364-0.718)
P6	Insomnia	0.421 (0.312-0.583)
P7	Insomnia	0.402 (0.282-0.572)
P5	Insomnia	0.397 (0.271-0.539)
P2	Insomnia	0.377 (0.268-0.536)
P3	Insomnia	0.349 (0.253-0.514)
P1	Insomnia	0.327 (0.231-0.483)
I6	IGD	0.268 (0.182-0.409)
I8	IGD	0.191 (0.103-0.334)
I9	IGD	0.179 (0.094-0.318)
P4	Insomnia	0.172 (0.090-0.313)
I7	IGD	0.129 (0.064-0.281)
I3	IGD	0.054 (0.011-0.153)
I5	IGD	0.047 (0.007-0.144)
I4	IGD	0.029 (0.002-0.101)

### Network Stability and Accuracy

The network model revealed 3 distinct variable clusters (Figure 1): the PSQI insomnia factors, the IGD symptom cluster, and SJL. The connection between the insomnia cluster and the IGD cluster was primarily bridged by I2 (withdrawal) as the key bridge variable. The connection weight between P7 and I2 was 0.329 (95% CI 0.246-0.450), indicating a strong positive association. Other insomnia factors (eg, P3, P5, and P6) also showed relatively high connection weights with I2 in IGD (P3: 0.155, 95% CI 0.081-0.305; P5: 0.313, 95% CI 0.225-0.456; P6: 0.186, 95% CI 0.043-0.3), while the remaining connection weights were all <0.15. Connections between the insomnia cluster and the SJL cluster were concentrated on P3 (0.240, 95% CI 0.131-0.384) and P5 (0.028, 95% CI −0.06 to 0.182). P2 and S1 formed a strong negative connection with a weight of −0.006 (95% CI −0.154 to 0.098). All connection weights between the insomnia factors and S1 were <0.3. The connections between the IGD cluster and the SJL cluster were exclusively facilitated by I6 (0.182, 95% CI 0.012-0.364), I7 (0.046, 95% CI 0.014-0.132) and I9 (0.033, 95% CI 0.009-0.121). The total connection weight among the 3 clusters was much higher than the total cross-cluster connection weight between insomnia and IGD or between insomnia and SJL, and no individual connection weight was >0.2.

**Figure 3:**
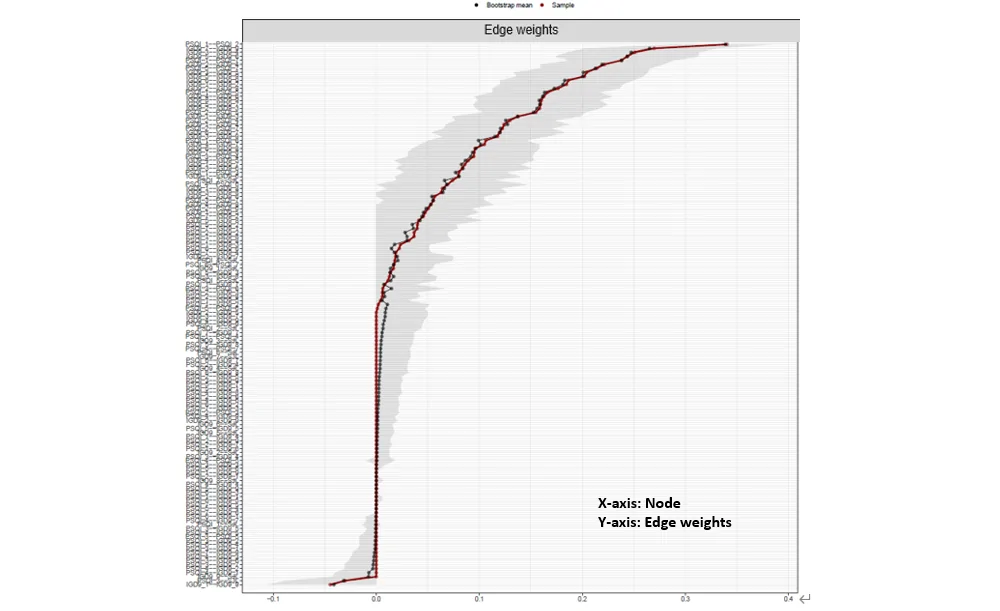
Bootstrapped CIs of edge weights. The red line indicates the edge weight values, and the gray area indicates the 95% CIs. IGD: internet gaming disorder; PSQI: Pittsburgh Sleep Quality Index; SJL: social jetlag.

The analysis showed that the CS coefficient of the model was 0.59, indicating that the indices were within an acceptable range. The bootstrap results demonstrated that the 95% CIs for the edge weights were relatively narrow, suggesting that the network exhibited acceptable accuracy (Figure 3).

## Discussion

### Principal Findings

This study aimed to identify the central and bridge symptoms within the comorbidity network of insomnia, IGD, and SJL. The detection rates among college students were 40.56% for insomnia, 18.28% for IGD, and 64.04% for significant SJL. Over the past decade, insomnia has been the most common sleep problem among college students in China, with an overall prevalence rate of 52.1% [30]. Chronic insomnia often leads to excessive use of mobile phones and the internet among students. In the Chinese university student population, the detection rate of IGD typically ranges from 5.4% to 20.9% [31], which aligns with our findings and is generally higher than that observed in adolescent populations. P1 and I9 occupied the most central positions within the entire network, indicating that they may play a critical role in maintaining and exacerbating the comorbid state of insomnia and IGD. Moreover, the network clearly revealed the interrelationships among the 3 symptom clusters, with I2 serving as the primary bridge connecting the insomnia and IGD clusters. These findings go beyond traditional correlation analyses by intuitively delineating the core role of specific symptoms within the comorbidity network, thereby providing precise entry points for subsequent mechanistic elucidation and targeted interventions.

Within the insomnia symptom cluster, subjective sleep quality (P1) demonstrated high strength centrality, indicating that an individual’s subjective evaluation of sleep quality had the strongest direct connections with other symptoms. As a central symptom, the centrality of subjective sleep quality is consistent with findings from a network analysis of sleep quality and sleep consciousness among Chinese adults, which also identified it as a central symptom [32]. The cognitive model of insomnia supports this perspective, proposing that excessive worry and catastrophizing about sleep constitute the core mechanisms underlying insomnia [33]. Individuals do not merely react to insufficient sleep itself, but rather to their perceived lack of sleep and its anticipated consequences. This sleep-related anxiety can trigger both emotional distress and physiological arousal, thereby perpetuating a vicious cycle [34]. When individuals subjectively perceive that they have slept poorly (ie, higher P1 scores), even in the presence of relatively adequate sleep duration (P3), such negative sleep appraisal alone may elicit intense negative emotions, such as frustration, irritability, and helplessness [35,36]. To escape these aversive nocturnal emotions, individuals may become more inclined to engage in online gaming [37]. The immersive environment and immediate rewards provided by gaming can temporarily divert attention away from sleep-related concerns, serving as a form of emotional avoidance and cognitive disengagement. This behavioral pattern—using online activities to cope with nocturnal anxiety and fill periods of wakefulness—aligns with the concept of compensatory internet use, forming a self-perpetuating loop [37]. Accordingly, the central role of P1 in the network highlights a potential key mechanism linking insomnia and IGD: escapist gaming behavior may be driven not by the objective loss of sleep per se but by the subjective catastrophizing interpretation of sleep disturbance and its accompanying emotional distress. This insight carries significant clinical implications. For individuals with comorbid insomnia and IGD, cognitive behavioral therapy for insomnia (CBT-I) should extend beyond traditional components, such as sleep restriction and stimulus control, to emphasize the modification of dysfunctional beliefs and attitudes about sleep. Such a cognitive restructuring approach may fundamentally reduce the motivational basis for emotion-driven gaming as a compensatory coping strategy.

The IGD symptom I9 emerged as the most central node in both strength and closeness centrality, indicating that impairment in social functioning and relationships exerts a profound influence within the IGD cluster and efficiently connects with other symptoms across the network. When individuals become engrossed in online gaming, their heightened pursuit of in-game achievements diminishes their capacity to control gaming time and behavior [38], consequently leading to problems associated with loss [39]. This phenomenon may also be explained by the concept of delay discounting [40]. Delay discounting refers to a decision-making bias in which individuals tend to devalue larger but delayed rewards in favor of smaller, immediate ones. The virtual world of online gaming constitutes an environment rich in immediate reinforcement (eg, leveling up, obtaining virtual items, and completing tasks), where rewards are both salient and easily attainable. In contrast, rewards in real-life domains, such as academic achievement, are inherently delayed and require sustained effort over time. Consequently, students may subjectively perceive the immediate gratification obtained through gaming as more valuable than delayed real-world rewards [14]. Accordingly, the symptom I9 may be reconceptualized as the direct behavioral manifestation of a series of shortsighted decisions driven by this cognitive bias. Such decision-making pathology contributes to a self-perpetuating vicious cycle: excessive gaming leads to real-life “losses” (eg, academic failure and relationship breakdown) [41], which—according to the interaction of person-affect-cognition-execution (I-PACE) model—exacerbate negative emotional states such as guilt, shame, and stress [42]. In turn, individuals may engage in compulsive gaming as a means of emotional compensation and avoidance, further reinforcing the addictive loop and consolidating the centrality of “loss” within the psychological and behavioral network. This mechanism suggests that university students with IGD may increasingly rely on gaming as an emotion regulation strategy [37]. From a clinical standpoint, this framework highlights that interventions should not merely address “loss” as a downstream outcome but rather target it as a central intervention node by modifying its underlying cognitive mechanisms [43]. Cognitive training approaches such as episodic future thinking (EFT) [44]—which enhance the mental representation of future outcomes and their subjective value—have been shown to effectively reduce delay discounting. Integrating such decision-making retraining modules into traditional cognitive behavioral therapy (CBT) protocols may help individuals more effectively evaluate the long-term consequences of their choices, thereby disrupting the core self-reinforcing loop that sustains IGD.

The connection between insomnia and the IGD symptom cluster is highly dependent on I2 as a key bridge node, with P3, P5, P6, and P7 all showing strong connection weights with the withdrawal symptom of IGD, suggesting that these pathways are key mechanisms underlying the comorbidity between insomnia and IGD [14]. Withdrawal, as a key diagnostic feature of addictive behaviors, is highly consistent with the interaction mechanism between gaming addiction and sleep disturbances. When individuals with IGD stop or reduce gaming, they experience withdrawal reactions such as irritability, inattention, and anxiety, which themselves disrupt sleep architecture, leading to sleep disturbances or daytime fatigue [45]. Moreover, the relationship is bidirectional: chronic sleep problems also impair emotional regulation and attention, making it more difficult for individuals to tolerate the discomfort associated with reduced gaming time, thereby forming a cyclical pattern between gaming withdrawal and sleep issues [46]. Studies have found that the hippocampus and caudate nucleus in the brain mediate the functional connectivity between insomnia and IGD severity. Given that the hippocampus is associated with emotion and the caudate nucleus with reward motivation, this may underlie the relationship between gaming withdrawal and sleep disturbances [47]. Alternatively, sleep disturbances may affect prefrontal inhibitory control and limbic emotional regulation, making individuals more sensitive to the negative emotions triggered by withdrawal and prompting them to increase internet use to alleviate discomfort, thus forming a vicious cycle [48]. Chronic sleep insufficiency can be viewed as sleep deprivation, which may alter reward pathway activity and the stress system, increasing sensitivity to internet-related cues and amplifying withdrawal discomfort [49]. Regarding daytime sleepiness, excessive daytime sleepiness and fatigue reduce real-world adaptability, driving individuals to seek compensation in virtual environments, while further impairing nighttime sleep and reinforcing the bidirectional interaction between addiction and insomnia [50]. Notably, P6 also showed a strong connection with I2, suggesting that pharmacological sleep interventions may play a unique role in the comorbidity of insomnia and IGD. Although long-term use of hypnotic medications may improve sleep in the short term, it can lead to abnormal sleep architecture, daytime sleepiness, and dependence, potentially exacerbating reward system imbalance and impulsivity, thereby reinforcing IGD withdrawal symptoms [48,51]. This finding suggests that in clinical practice, for patients with insomnia and a tendency toward IGD, hypnotic medications should be used with caution, and nonpharmacological interventions such as CBT should be prioritized to avoid reinforcing addiction-related pathways [47].

The direct connection weights between SJL and both “continue despite problems” and “deception” were low, yet certain correlations remained. This suggests that SJL may not be directly associated with these symptoms but rather be linked indirectly through mediating pathways such as reduced sleep duration or impaired cognitive function. From the perspective of network theory, “continue despite problems” may act as a critical bridge symptom, reflecting a failure of metacognitive control in which individuals are unable to effectively suppress their urge to engage in gaming despite being aware of its negative consequences [19]. Previous studies have shown that the association between SJL and addictive behaviors can be mediated by mechanisms such as attentional allocation, emotional state, and time perception bias [52]. The results of the present network analysis further suggest that such mediating pathways may ultimately converge on the cognitive node of knowing transgression. “Continue despite problems” may also contribute to the formation of addictive behaviors and, in turn, exacerbate SJL. When cognitive function is impaired, individuals may struggle to weigh the immediate rewards of gaming against long-term goals in real life, thereby increasing the likelihood of abandoning important activities or even engaging in deceptive behaviors. The neural mechanisms associated with reward processing may underlie the link between SJL and “deception” in online gaming. The reward system serves to organize behavior in the context of rewarding outcomes, and the likelihood of obtaining rewards varies with the light-dark cycle [53]. Consequently, the circadian system may be altered, leading to changes in SJL, potentially due to reduced responsiveness of the medial prefrontal cortex to reward anticipation and receipt [54]. Meanwhile, SJL and circadian disruption have been found to alter reward sensitivity. Evening chronotypes, who typically exhibit greater SJL, show a heightened preference for immediate rewards and reduced sensitivity to delayed punishments [55]. Such neurocognitive changes may render individuals more susceptible to immersion in game achievement systems while neglecting real-life responsibilities, ultimately exacerbating the symptom of “deception” [56].

Withdrawal serves as the strongest bridge node in the current network model of IGD, SJL, and insomnia. Therefore, targeting this key pathway associated with withdrawal symptoms may effectively interrupt the vicious cycle between insomnia and IGD, making it an important nonpharmacological intervention strategy for addressing their comorbidity. Considering the connection patterns between SJL and the symptoms of “continue despite problems” and “deception,” SJL can be regarded as a key intervention target. Time-based therapies (eg, timed light exposure and rhythmic melatonin supplementation) can be used to synchronize individual circadian rhythms with social schedules, complemented by sleep hygiene education to stabilize sleep duration and reduce sleep variability. For individuals who meet the diagnostic criteria for IGD, routine assessment of sleep architecture and withdrawal symptom severity should be conducted alongside targeted interventions for addictive behaviors. By improving sleep disturbances and enhancing sleep continuity, these interventions are expected to alleviate the cognitive and emotional dysregulation mediated by sleep deprivation, thereby reducing IGD withdrawal symptoms and achieving synergistic improvements in both insomnia and IGD.

### Limitations

Despite its contributions, this study has limitations. Its cross-sectional design precludes causal inferences regarding the directionality of the identified relationships. Future research should use longitudinal designs or experience sampling methods to track the temporal dynamics of this network. Although participants in this study were recruited offline, the questionnaires were uniformly administered online, without a control sample completing paper-and-pencil versions. Future research could incorporate samples using both administration formats to examine the potential impact of data collection modality on the results and thereby enhance the applicability of the findings. Moreover, as the sample consisted of nonclinical participants, the generalizability of this network structure to clinical populations and other cultural contexts requires further validation. Future studies should also incorporate objective sleep measures, such as actigraphy, and explore the roles of other variables, such as negative affect and cognitive control, within this network. Finally, this study did not consider variations in the educational background of the middle schools from which the college students graduated (eg, academic pressure and management styles), factors that may potentially influence their sleep rhythms and gaming behaviors. Future studies should include middle-school–level variables for more comprehensive analyses.

### Conclusions

This study is the first to apply network analysis to simultaneously examine the symptom structure of IGD, insomnia, and SJL in a large sample of Chinese college students. Unlike previous studies that typically focused on bivariate associations or single disorders, our findings reveal that P1 and I9 are central nodes within their respective communities, while I2 serves as the key bridging symptom connecting insomnia and IGD. I2 acts as the primary bridge linking the insomnia and IGD clusters, which strongly supports the cyclical theory of emotional effects: individuals’ withdrawal reactions lead to sleep disturbances, and sleep disturbances, in turn, contribute to emotional problems that exacerbate withdrawal symptoms. Therefore, subsequent interventions should prioritize targeting withdrawal symptoms and sleep dissatisfaction to simultaneously alleviate comorbid symptoms. The role of SJL was relatively limited, suggesting that its impact on IGD is likely indirect. This implies that managing circadian rhythm disruption may indirectly reduce the risk of IGD. Collectively, this study provides actionable symptom targets for prevention and treatment programs in university settings.

## Abbreviations

CBT: cognitive behavioral therapy
CBT-I: cognitive behavioral therapy for insomnia
CS: correlation stability
EFT: episodic future thinking
GGM: Gaussian graphical model
ICD-11: International Statistical Classification of Diseases and Related Health Problems, 11th edition
IGD: internet gaming disorder
I-PACE: Interaction of Person-Affect-Cognition-Execution
IRB: institutional review board
JASP: Jeffreys’ Amazing Statistics Program
LASSO: least absolute shrinkage and selection operator
MCAR: missing completely at random
MCTQ: Munich Chronotype Questionnaire
MOBA: multiplayer online battle arena
MSF: midsleep time on free days
MSW: midsleep time on workdays
PSQI: Pittsburgh Sleep Quality Index
SJL: social jet lag
WJX: Wenjuanxing
